# Morphological remodeling of canine lymphocytes in *Ehrlichia canis* infection: quantitative analysis by scanning electron microscopy and fractal dimension

**DOI:** 10.1590/S1984-29612026021

**Published:** 2026-07-06

**Authors:** Caroline Pedroso de Oliveira, Marcela Aldrovani Rodrigues, Maysa Barbosa de Almeida, Caio Rafael Siqueira Vasconcelos, Elisângela Rosa da Silva, Rafael Paranhos de Mendonça, Fernanda Gosuen Gonçalves Dias

**Affiliations:** 1 Universidade de Franca – UNIFRAN, Programa de Pós-graduação em Ciência Animal, Franca, SP, Brasil; 2 Universidade Federal de Uberlândia – UFU, Instituto de Ciências Biológicas, Departamento de Biofísica, Uberlândia, MG, Brasil

**Keywords:** Canine ehrlichiosis, lymphocyte morphology, cell membrane, scanning electron microscopy, fractal analysis, immune activation, Erliquiose canina, morfologia de linfócitos, membrana celular, microscopia eletrônica de varredura, análise fractal, ativação imunológica

## Abstract

This study aimed to quantitatively assess morphological surface changes in lymphocytes from dogs naturally infected with*Ehrlichia canis*using scanning electron microscopy (SEM), fractal dimension analysis, and nucleus-to-cytoplasm (NC) ratio determination. Thirty dogs (infected group, n=15; control group, n=15) underwent blood count, blood smear analysis, rapid diagnostic testing, and polymerase chain reaction confirmation. Mononuclear cells were isolated by density gradient centrifugation and processed for imaging. Images were analyzed for morphometric measurements and pseudocoloring. The infected group exhibited higher proportions of lymphocytes with medium (44.4%) and low (13.3%) NC ratios compared to controls (22.2% and 8.9%, respectively), indicating cellular activation (*p* = 0.03). SEM revealed marked reduction in surface protrusions in infected lymphocytes. Morphometric analysis showed no differences in cell length, width, diameter, or circumference area between infected and control group; however, lymphocytes of the infected group displayed greater radius and circumference length (*p* < 0.05). Fractal dimension values showed no differences between groups (*p* > 0.05). The integrated approach combining SEM, morphometry, and fractal analysis quantified lymphocyte morphological alterations associated with *E. canis* infection, reflecting cellular activation and maturation. These findings advance understanding of immunopathological mechanisms in canine ehrlichiosis and highlight the potential of advanced morphometric techniques in immunopathology.

## Resumo

Este estudo avaliou quantitativamente as alterações morfológicas de superfície de linfócitos em cães naturalmente infectados por*Ehrlichia canis*utilizando microscopia eletrônica de varredura (MEV), análise de dimensão fractal e determinação da razão núcleo-citoplasma (NC). Trinta cães (grupo infectado, n=15; grupo controle, n=15) foram submetidos a hemograma, esfregaço sanguíneo, teste rápido e confirmação por PCR. Células mononucleares do sangue periférico foram isoladas por centrifugação em gradiente de densidade e processadas para MEV. Imagens foram analisadas para medidas morfométricas e pseudocoloração. O grupo infectado exibiu maiores proporções de linfócitos com razões NC média (44,4%) e baixa (13,3%) comparado aos controles (22,2% e 8,9%), indicando ativação celular (*p* = 0,01). A MEV revelou redução marcante nas projeções de superfície. A análise morfométrica não mostrou diferenças em comprimento, largura, diâmetro ou área; entretanto, linfócitos infectados exibiram maior raio e comprimento de circunferência (*p* < 0,05). A dimensão fractal não diferiu entre os grupos (p > 0,05). A abordagem integrada combinando MEV, morfometria e análise fractal quantificou as alterações morfológicas de linfócitos associadas à infecção por *E. canis*, refletindo ativação e maturação celular. Estes achados avançam a compreensão dos mecanismos imunopatológicos da erliquiose canina e destacam o potencial das técnicas morfométricas avançadas na veterinária.

## Introduction

Canine ehrlichiosis, caused by the rickettsial bacterium*Ehrlichia canis*, is a tick-borne infectious disease with high prevalence in tropical and subtropical regions, representing a significant veterinary challenge due to its complex immunopathological mechanisms and variable clinical manifestations (Rodríguez-Alarcón et al., 2020; Castro et al., 2022). The infection primarily targets circulating lymphocytes and monocytes -serving as pathogen reservoirs - triggering systemic inflammatory responses that compromise hematologic parameters and immune function (Al-Dulaimi et al., 2021). Despite the clinical relevance of canine ehrlichiosis, the morphological dynamics of lymphocyte activation during*E. canis*infection remain incompletely understood.

Lymphocytes play a central role in the adaptive immune response against intracellular pathogens, with their morphology reflecting activation status, maturation stage, and functional capacity (Hang et al., 2017; Al-Dulaimi et al., 2021). Conventional diagnostic methods, such as blood smears and hematologic analysis, provide limited morphological information, necessitating more sophisticated approaches to characterize surface-related changes associated with immune activation (Passey et al., 2007). Lymphocyte surface architecture is directly associated with essential immune functions -including antigen recognition, cell signaling, and immunological synapse formation - that conventional hematological or molecular analyses fail to capture (Passey et al., 2007). Scanning electron microscopy (SEM) offers high-resolution imaging of cellular surface structures, particularly microvilli and membrane protrusions involved in cell-cell interactions (Passey et al., 2007; Orbach & Su, 2020). However, comprehensive quantitative morphometric assessments integrated with SEM remain scarce in the context of rickettsial infections in veterinary medicine. While hematological and immunophenotypic alterations in canine ehrlichiosis are relatively well described, studies providing quantitative high-resolution characterization of lymphocyte surface remodeling during *E. canis* infection are lacking (Orbach & Su, 2020).

Fractal dimension analysis, a mathematical approach to quantify surface complexity and irregularity (Buruiană et al., 2024), has emerged as a sensitive tool for assessing morphological changes in various pathological conditions (Xavier et al., 2018; Wu et al., 2020; Mah et al., 2022; Battalapalli et al., 2023). The combination of SEM, quantitative morphometry, and fractal analysis provides an integrated platform to detect and characterize cellular alterations associated with infection. To date, few studies in veterinary medicine have combined ultrastructural imaging with quantitative morphometric and fractal analysis to evaluate immune cell morphology, underscoring the novelty and relevance of this integrated approach (Xavier et al., 2018).

Despite these advances, it remains unclear whether lymphocytes from dogs naturally infected with*E. canis*exhibit quantifiable structural and surface alterations -detectable via such methods - that may reflect changes in immune cell behavior. Therefore, this study aims to quantitatively assess morphological surface changes in lymphocytes from dogs naturally infected with*E. canis*using SEM, fractal dimension calculations, and nucleus-to-cytoplasm (NC) ratio analysis, to elucidate immune cell dynamics and activation patterns during canine ehrlichiosis.

## Material and Methods

### Ethical aspects and animal inclusion

This research was approved by the Animal Ethics Committee (CEUA) of the University of Franca (UNIFRAN), under protocol number 3021120723. Thirty dogs were prospectively enrolled from the clinical caseload of the UNIFRAN Veterinary Hospital, comprising 15 animals per group: the *Ehrlichia* group (EG) and the control group (CG). Dogs of both sexes, of different ages and breeds, and weighing >10 kg were included after standardized physical examination to investigate lymphocyte morphological alterations associated with naturally occurring *E. canis* infection.

Inclusion criteria were as follows: for the EG, clinical suspicion of canine monocytic ehrlichiosis based on compatible signs (fever, lethargy, anorexia, lymphadenomegaly, or hemorrhagic manifestations), a positive SNAP® 4Dx Pro rapid test (IDEXX Laboratories, USA), confirmatory PCR for *E. canis*, and a characteristic hematologic profile, including thrombocytopenia and regenerative anemia; for the CG, absence of clinical signs consistent with tick-borne disease, negative diagnostic tests, and normal hematologic parameters. Detailed diagnostic procedures are described in the “Blood collection and initial screening” section.

Exclusion criteria included a history of neoplasia or autoimmune disease; administration of antibiotics or immunomodulatory drugs within 30 days before enrollment; multiplex PCR-confirmed coinfections with *Anaplasma* spp., *Babesia* spp., or *Hepatozoon* spp.; severe pre-existing hematologic disorders unrelated to ehrlichiosis (e.g., bone marrow failure syndromes or hemolytic anemia); and extreme hematologic abnormalities inconsistent with ehrlichiosis (e.g., WBC <3.000/μL or platelet count <20.000/μL). Other clinically relevant canine pathogens were excluded based on clinical history and routine diagnostic findings, including negative endemic screening for *Leishmania* spp., absence of evidence suggestive of *Bartonella* spp. infection (e.g., flea infestation or endocarditis), and normal monocyte counts, considered inconsistent with *Mycoplasma haemocanis* infection.

Dogs were consecutively recruited from the clinical caseload over a 6-month period, yielding a convenience sample of 15 animals per group. This sample size was estimated to provide >80% statistical power (α = 0.05; unpaired *t*-test) to detect moderate-to-large effect sizes (Cohen’s *d* > 0.8) in the primary morphometric outcomes, such as differences in the NC ratio, consistent with exploratory studies of similar design in veterinary immunopathology.

### Blood collection and initial screening

Dogs presenting to the UNIFRAN Veterinary Hospital clinical routine underwent initial physical examination. Blood collection was performed via venipuncture of the external jugular vein using sterile BD PrecisionGlide® syringes (Becton Dickinson, Curitiba, Brazil) from all animals meeting preliminary clinical criteria. Aliquots were stored in microtubes containing ethylenediaminetetraacetic acid (EDTA; Analisa, Belo Horizonte, Brazil) and sent to the UNIFRAN Veterinary Hospital Clinical Laboratory.

Complete blood counts were performed using an automated hematology analyzer (pocH-100iv Diff; Sysmex, São José dos Pinhais, PR, Brazil) following classical techniques (Thrall et al., 2014). Blood smears were prepared on frosted-edge glass slides (Knittel, Braunschweig, Germany), stained using a rapid panoptic method (Instant Prov Kit; Newprov), and analyzed under optical microscopy (Leica Microsystems DMLB; Wetzlar, Germany) at 1000x magnification to identify intracellular morulae of *E. canis* and determine the nucleus-to-cytoplasm (NC) ratio of lymphocytes.

To ensure representative sampling and minimize bias, lymphocyte selection followed standardized criteria: cells were selected from the smear monolayer region (excluding peripheral accumulation zones), with size classification relative to adjacent erythrocytes (7-8 μm diameter) per veterinary hematology standards (Thrall et al., 2014) - small (~RBC size; 7-10 μm), medium (1.2-1.5× RBC; 10-12 μm), large (>1.5× RBC; ≥12-15 μm) - measured via ImageJ (National Institutes of Health, Bethesda, MD, USA) on digital images (n=15 cells/category/group; total n=45 cells/group).

### Nucleus-to-cytoplasm (NC) ratio analysis

The sample size of 45 lymphocytes per group (infected group and control group) was defined based on standardized morphometric image-analysis protocols, ensuring adequate representation of the lymphocyte population while accounting for natural size variation and allowing reliable statistical comparison between groups (Table 1). Representative lymphocyte images were obtained from blood smears using a coupled digital camera, following the selection criteria described above (monolayer region, well-preserved morphology, absence of compression artifacts), with a stratified approach to include proportional numbers of small, medium, and large lymphocytes (n=15 cells per size category per group).

**Table 1:** Observed absolute and relative frequencies of lymphocytes distributed according to nucleus-to-cytoplasm (NC) ratio categories in dogs naturally infected with *Ehrlichia canis* (*Ehrlichia* group - EG) and control dogs (CG).

**NC ratio category**	***E. canis* (EG), n (%)**	**Control (CG), n (%)**	**χ^2^**	***p***
High (>0.41)	19 (42.2)	31 (68.9)	6.61	0.037
Medium (0.21-0.41)	20 (44.4)	10 (22.2)		
Low (<0.21)	6 (13.3)	4 (8.9)		

Comparisons between groups were performed using a chi-square test of independence applied to the full 3 × 2 contingency table. The test evaluates the overall association between NC ratio categories and experimental groups (*Ehrlichia* and control) and does not provide pairwise comparisons between individual categories. Values are expressed as n (%).

The NC ratio was determined using digital image processing, adapted from Hang et al. (2017). Grayscale images were analyzed in ImageJ software; nuclear contours were manually delineated using the Polygon Selection Tool, and both nuclear area and total cell area were measured. The NC ratio was calculated as nuclear area divided by cytoplasmic area (NC ratio = nuclear area / [total cell area – nuclear area]). Lymphocytes were then classified into three NC ratio categories: high (>0.41), medium (0.21-0.41), and low (<0.21). The relative frequency of each category was determined for both groups.

### Diagnostic testing and group assignment

Sera from suspected ehrlichiosis cases were tested using SNAP® 4Dx Plus rapid test (IDEXX Laboratories, USA) following manufacturer recommendations. Dogs positive for *E. canis* were assigned to the ehrlichiosis group (EG, n=15); negative dogs to the control group (CG, n=15). Among animals in the EG, clinical assessment was performed to characterize disease manifestations: cases were classified as symptomatic (n= 15) if presenting clinical signs consistent with ehrlichiosis (including fever, lethargy, anorexia, lymphadenomegaly, or hemorrhagic manifestations) or asymptomatic (n= 0) if no clinical signs were evident at the time of blood collection.

Confirmation of *E. canis* monoinfection and exclusion of major tick-borne coinfections was performed via polymerase chain reaction (PCR). DNA was extracted from 200 μL EDTA-blood using UltraClean™ DNA BloodSpin Kit (Mo Bio Laboratories, Inc., Carlsbad, CA, USA). Multiplex PCR targeted *Anaplasma* spp., *Babesia* spp., *E. canis*, and *Hepatozoon* spp. using previously validated primers (Felek et al., 2003; Götsch et al., 2009; Almeida et al., 2012). Animals with any coinfection were excluded.

### Isolation and preparation of peripheral blood mononuclear cells

Peripheral blood mononuclear cells (PBMCs) were isolated by density gradient centrifugation using Histopaque 1077 (Sigma-Aldrich, St. Louis, MO, USA) as previously described (Vasconcelos et al., 2025). Blood samples were diluted 1:1 with sterile, isotonic phosphate-buffered saline (PBS) and carefully layered over Histopaque 1077. Samples were centrifuged at 700 g for 20 minutes at 25°C with minimal acceleration/deceleration. The mononuclear cell layer at the plasma-Histopaque interface was aspirated, washed in PBS, and fixed in Karnovsky's solution for SEM analysis.

### Scanning Electron Microscopy (SEM)

PBMC pellets were removed from Karnovsky's solution, washed in PBS, and dehydrated in increasing concentrations of ethyl alcohol. Using an automatic pipette, 2 µL of dehydrated pellets were placed on glass coverslips (Knittel) and air-dried at room temperature for 30 minutes to promote cell adhesion. Coverslips were incubated at 60°C for 5 hours, cooled, and fixed onto metal stubs using carbon tape. At the Electron Microscopy Laboratory of the Federal University of Uberlândia, coverslips were coated with 30 nm of gold-palladium using a Sputter Coater SCD (Bal-Tec, Germany) following published guidelines (Czerwińska-Główka & Krukiewicz, 2021). Images were acquired using a scanning electron microscope (TESCAN Vega 3SBH Easy Probe; resolution 3 nm; 30 kV tungsten filament) equipped with a backscattered electron detector (Oxford Instruments x-act), capturing five images per sample at standardized magnification (scale bar = 5 µm) using VegaTC operating software. This magnification level (approximately 10.000 x) was selected to provide sufficient resolution for visualization and quantification of surface structures, particularly microvilli and membrane protrusions, while maintaining adequate field coverage for morphometric analysis.

### Morphometric analysis

Morphometric parameters were recorded from the SEM images using VegaTC software (Tescan, Czech Republic), including lymphocyte length, width, radius, diameter, circumference length, and circumference area. To ensure comparable and representative analysis between groups, the following criteria were applied: only cells with clear membrane definition and minimal fixation artifacts were selected for analysis; consistent methodology was maintained across all samples, with morphometric measurements performed on n=30 lymphocytes per group (representing approximately two lymphocytes per animal within each size category); cells were selected proportionally from each lymphocyte size category to account for natural population heterogeneity; and measurements were conducted in a blinded manner by trained personnel to minimize observer bias. These standardized procedures ensured that morphometric comparisons between the EG and CG were based on equivalent and representative lymphocyte populations, allowing valid assessment of infection-related morphological alterations. Additional pixel-based measurements (perimeter, Feret's ratio, asymmetry, and kurtosis) were obtained using ImageJ for statistical comparison.

### Fractal dimension analysis

Fractal dimension was calculated using the box-counting method (Xavier et al., 2018; Wu et al., 2020; Mah et al., 2022) to quantify surface irregularity and membrane complexity. Consistent with the morphometric analysis methodology, fractal dimension calculations were performed on n=30 lymphocytes per group, selected proportionally from each lymphocyte size category to ensure population representativeness. For each cell, the fractal dimension was obtained from the slope of a regression line generated by plotting the logarithm of box count (y-axis) against the logarithm of box size (x-axis), using the scaling range determined by the pixel dimensions of the cell structure. Analysis was conducted using ImageJ with the Fraclac plugin (version 2.5), with standardized parameters applied uniformly across all samples. The box-counting method provides a scale-invariant measure of membrane complexity that is particularly sensitive to changes in cellular architecture.

### Statistical analysis

Statistical analyses were performed using GraphPad Prism 8.0 software (GraphPad Software, San Diego, CA, USA). Continuous variables were analyzed using unpaired t-tests with Welch’s correction for unequal variances. For categorical data (NC ratio classification: high, medium, and low), a chi-square test of independence was applied to the full contingency table (3 × 2) to assess the association between NC ratio distribution and experimental group (*Ehrlichia* group vs. control group). No post hoc pairwise comparisons between categories were performed. Statistical significance was set at *p* < 0.05.

## Results

### Complete blood count

Complete blood counts confirmed classic hematologic alterations of canine ehrlichiosis. The *Ehrlichia* group (EG) showed regenerative anemia (RBC: 4.3±1.3× 10^6^/μL; Hb: 8.8±2.5 g/dL; Hct: 27.7±7.96%) and thrombocytopenia (91,137.5 ±55,953.3/μL) compared to controls (RBC: 7.5±1.0 × 10^6/^μL; Hb: 16.6±1.7 g/dL; Hct: 49.8±5.1%; platelets: 316,626.7±102,880.1/μL). Leukocytosis was evident in EG (13,931.3±10,004.1/μL) versus controls (10,626.7±2,653.7/μL), characterized by neutrophilia (segmented: 11,538.8±9,734.01/μL; bands: 446.2±232.72/μL) and relative lymphopenia (1.602.7±1.163.4/μL vs. 2,583.1±1.349.0/μL). Monocytes (247.0± 163.8/μL) and basophils (595.6±450.0/μL) showed similar counts between groups.

### Blood smear and NC ratio analysis

The NC ratio categorization showed a significant overall association between groups (χ^2^ = 6.61, p = 0.037). Descriptively, lymphocytes with high NC ratio were more prevalent in the control group, whereas medium and low NC ratios were more frequent in the infected group. Lymphocytes with high NC ratio were observed in 31/45 (68.9%) of the control group and 19/45 (42.2%) of the infected group. Conversely, medium NC ratios were identified in 20/45 (44.4%) of the infected group and 10/45 (22.2%) of controls, while low NC ratios accounted for 6/45 (13.3%) and 4/45 (8.9%), respectively.

### SEM: surface protrusions and morphometric analysis

**Figure 1:**
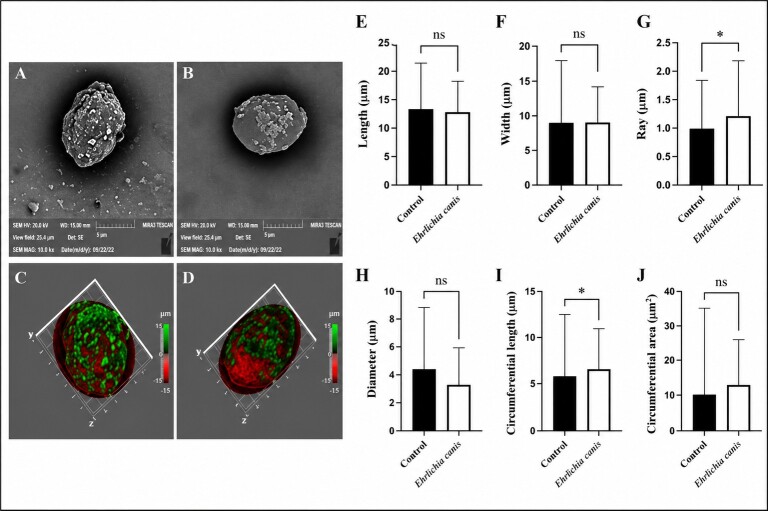
Scanning electron microscopy: surface protrusions and morphometric analysis of lymphocytes in canine ehrlichiosis. (A, B) Scanning electron microscope images of lymphocytes from a control dog (A) showing abundant surface microvilli protrusions compared to a dog naturally infected with*Ehrlichia canis*(B), demonstrating marked reduction in surface complexity; (C, D) Pseudocoloring and plotting analysis using ImageJ software confirming reduced microvilli density in infected animals (green overlay), with red indicating remaining cellular structures in both control (C) and infected (D) lymphocytes; (E-J) Morphometric measurements recorded using SEM software, comparing control (black bars) and*E. canis*-infected (white bars) lymphocytes for: (E) length, (F) width, (G) radius, (H) diameter, (I) circumference length, and (J) circumference area. Asterisks (*) indicate p < 0.05; ns indicates not significant (*p* > 0.05).

SEM images revealed marked reduction in surface protrusions on lymphocytes (Figure 1B) from EG dogs compared to CG dogs (Figure 1A). Pseudocoloring and plotting analysis using ImageJ confirmed reduced microvilli density in infected animals (Figures 1C and 1D). Measurements recorded using SEM software (Figures 1E-J) showed no significant differences between groups regarding lymphocyte length, width, diameter, or circumference area (*p* > 0.05). However, lymphocytes from EG displayed significantly greater radius (*p* < 0.05) and circumference length (*p* < 0.05) compared to CG.

### ImageJ-derived measurements: pixel-based morphometry and fractal dimension

**Figure 2:**
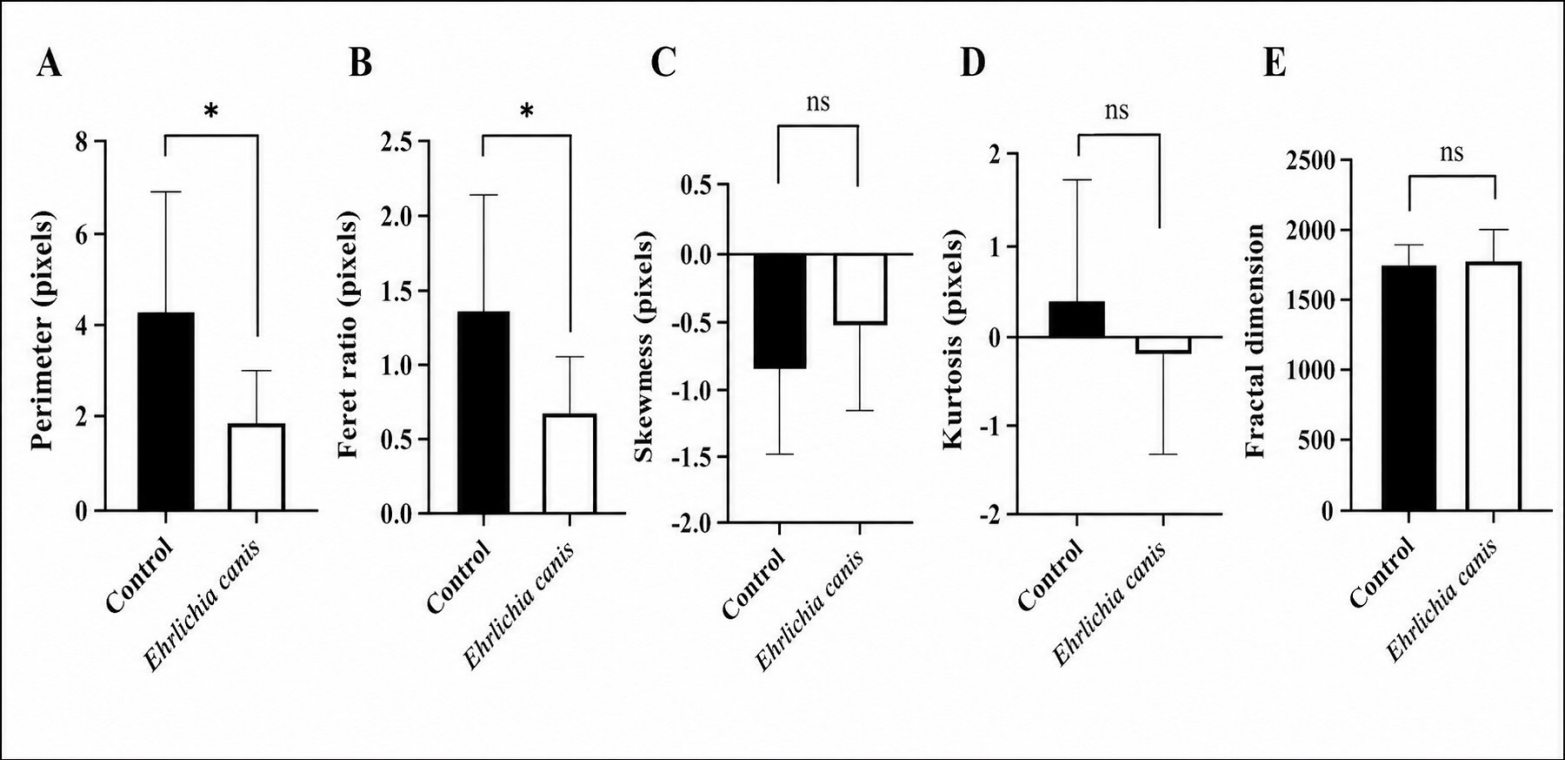
ImageJ-derived measurements: pixel-based morphometry and fractal dimension analysis of lymphocyte surfaces. (A-D) Pixel-based morphometric measurements comparing control (black bars) and*Ehrlichia canis*-infected (white bars) lymphocytes for: (A) perimeter, (B) Feret's ratio, (C) asymmetry, and (D) kurtosis. (E) Fractal dimension values calculated using ImageJ box-counting method. Asterisks (*) indicate *p* < 0.05; ns indicates not significant (*p* > 0.05).

Pixel-based measurements obtained using ImageJ software revealed significantly higher perimeter and Feret's ratio values in the CG compared to EG (*p* < 0.05; Figures 2A and 2B), while asymmetry and kurtosis showed no significant differences between groups (*p* > 0.05; Figures 2C and 2D). Fractal dimension values of lymphocyte surfaces, calculated using ImageJ box-counting method, showed no significant differences between EG and CG (*p* > 0.05; Figure 2E).

## Discussion

The present study demonstrated significant morphological alterations in lymphocytes from dogs infected with *E. canis*. Although no morulae were observed in monocytes or other blood cells, this finding does not rule out infection**,** since the detection of morulae in peripheral blood smears has low sensitivity and depends on parasitemia and the stage of infection. In subclinical or chronic infections, the number of circulating infected monocytes may be too low for reliable microscopic identification. Moreover, conventional blood smears are less sensitive than buffy coat preparations for detecting intracellular inclusions (Hang et al., 2017). Therefore, the absence of morulae was considered compatible with the PCR-confirmed diagnosis in this study.

PCR confirmation strengthened the diagnostic robustness of the study by ensuring high specificity for *E. canis* infection. In addition, the absence of coinfections among the included animals minimized potential confounding by other infectious agents, thereby supporting the association between the observed lymphocyte morphological alterations and *E. canis* infection (Almeida et al., 2012).

Lymphocytes play a central role in the immune response against infections, and their morphological characteristics may reflect structural remodeling associated with activation and maturation states, including variations in nucleus-to-cytoplasm ratio and membrane architecture, although these features do not directly demonstrate functional activation such as cytokine production or intracellular signaling (Hang et al., 2017; Al-Dulaimi et al., 2021). The NC ratio is a widely used morphological marker for assessing lymphocyte maturation and activation status, with immature or resting lymphocytes typically exhibiting high NC ratios and activated lymphocytes displaying reduced NC ratios due to increased cytoplasmic volume (Hang et al., 2017). In the present study, lymphocytes from *E. canis*-infected dogs showed significantly higher proportions of cells with medium and low NC ratios compared to control dogs, which is consistent with cellular activation and maturation during the immune response to infection (Hang et al., 2017; Al-Dulaimi et al., 2021). These observations are also in line with previous reports describing increased participation of CD8+ T lymphocytes in *E. canis* infection, a population involved in the elimination of infected cells and in the cellular immune response (Walker & Dumler, 2015; Castro et al., 2022). Taken together, these findings suggest that the shift toward lower NC ratios may reflect adaptive lymphocyte remodeling in response to an intracellular pathogen.

A notable finding of SEM analysis was the significant reduction in surface protrusions in lymphocytes from naturally infected dogs by *Ehrlichia canis*. Microvilli are dynamic physiological structures that extend the cell surface, facilitating interactions with other immune cells and antigen-presenting cells (Passey et al., 2007; Orbach & Su, 2020). These protrusions are involved in the formation of immunological synapses, particularly in T lymphocytes, where they contribute to the organization of surface receptors and signaling molecules required for cell-cell communication. However, as lymphocyte subpopulations were not distinguished in this study, these morphological findings should be interpreted as general features associated with lymphocyte interaction and activation processes, rather than being attributed to a specific lymphocyte subset or signaling pathway (Dustin & Cooper, 2000). Therefore, the reduction in surface protrusions observed in lymphocytes from *E. canis*-infected dogs may indicate altered cell-cell interaction capacity, potentially affecting antigen recognition and the efficiency of adaptive immune responses (Orbach & Su, 2020). In addition, surface protrusions are enriched in adhesion molecules and receptors involved in signaling, proliferation, migration, and adhesion to vascular endothelium (Kim & Jun, 2019). Accordingly, a reduction in microvilli may also compromise lymphocyte trafficking and recruitment to sites of immune activity, thereby contributing to dysregulation of the host response during rickettsial infection (Bagatini et al., 2018).

Morphometric analysis revealed that lymphocytes from *E. canis*-infected dogs displayed significantly greater radius and circumference length compared to those from the CG group, whereas cell diameter and circumference area remained unchanged.

This pattern suggests selective morphological remodeling rather than a uniform increase in overall cell size**,** and is consistent with activation-related changes described in other infectious conditions, including viral and bacterial diseases, in which lymphocyte shape and structural complexity are altered during immune stimulation (Novotney et al., 1990; Lerner et al., 1998; Zheng et al., 2018). The observation that some morphometric parameters were increased in infected lymphocytes, whereas perimeter and Feret’s ratio were higher in control cells, further supports the interpretation that the changes involved cell shape remodeling rather than simple enlargement or shrinkage. Similar asymmetric deformations have been reported in chronic infections, such as T cell activation during *Trypanosoma cruzi* infection (Mendes-da-Cruz et al., 2003). Moreover, the absence of significant differences in asymmetry and kurtosis suggests that, despite these alterations, the overall distribution of cell shape remained relatively balanced, which may indicate a regulated rather than a random remodeling process (Orbach & Su, 2020).

Fractal dimension analysis, a mathematical approach used to quantify surface complexity and irregularity, revealed no significant differences between *E. canis*-infected and control lymphocytes. Fractal dimension has been recognized as a sensitive indicator of morphological complexity and has been used to evaluate surface irregularities in various pathological conditions, including sepsis (Hohlstein et al., 2019; Naydenov et al., 2020). In the present study, the maintenance of similar fractal dimension values despite differences in other morphometric parameters suggests that global membrane complexity remained relatively stable. This finding may indicate that, although lymphocytes underwent measurable shape remodeling and loss of surface protrusions, these changes were not sufficient to alter the overall topological organization detected by box-counting analysis. Accordingly, the results support the interpretation that *E. canis* infection was associated with localized ultrastructural changes rather than a generalized disruption of membrane complexity.

The morphological changes observed in this study, including reduced NC ratio, increased circumference length, and decreased surface protrusion density, are consistent with patterns described in activated lymphocytes during immune responses to various infectious agents (Hang et al., 2017; Al-Dulaimi et al., 2021). Nevertheless, morphological alterations alone cannot be considered definitive evidence of lymphocyte activation**,** as such changes may reflect different functional states or nonspecific responses to infection**.** A more comprehensive characterization of lymphocyte activation would require complementary analyses, including the expression of activation markers such as CD25, CD69, and HLA-DR, assessment of cytokine production (e.g., IL-2, IFN-γ, and TNF-α), and evaluation of intracellular signaling pathways such as phosphorylated ERK1/2, p38-MAPK, and NF-κB. Accordingly, future studies integrating these approaches may help clarify whether the morphological alterations identified here are directly associated with specific activation pathways in lymphocytes during *E. canis* infection.

Another important consideration is whether the morphological alterations observed in this study are specific to canine ehrlichiosis or instead reflect a broader response to infectious disease. Lymphocyte morphological remodeling has been described in association with a variety of pathogens, including viruses, bacteria, fungi, and parasites. In the present study, the likelihood that these findings were associated with *E. canis* infection was strengthened by several design features, including PCR confirmation of infection, exclusion of concurrent infections with *Anaplasma* spp., *Babesia* spp., and *Hepatozoon* spp., systematic exclusion of animals with a history of other infections, neoplasia, or recent antibiotic or immunomodulatory treatment, and comparison with matched uninfected controls. Even so, the cross-sectional design precludes a definitive causal attribution to *E. canis*, since the observed lymphocyte alterations may represent secondary responses to infection rather than direct pathogen-specific effects**.** Longitudinal studies tracking animals from pre-infection to recovery, together with in vitro stimulation of lymphocytes with *E. canis* antigens, would be valuable to clarify the specificity and biological basis of these morphological changes.

## Conclusions

The integrated application of SEM, quantitative morphometry, and fractal dimension analysis proved useful for characterizing lymphocyte morphological remodeling in dogs naturally infected with *E. canis*. Overall, the findings support the interpretation that *E. canis* infection is associated with morphologic patterns compatible with lymphocyte activation and maturation, reinforcing the value of these approaches for investigating host immune responses in canine ehrlichiosis. Beyond documenting structural changes, this study highlights the potential of advanced morphometric tools to improve the investigation of immune cell dynamics in veterinary pathology. These results provide a basis for future studies aimed at clarifying the immunopathological mechanisms underlying canine ehrlichiosis and the broader applicability of quantitative ultrastructural analysis in veterinary medicine.

## Data Availability

The data supporting the findings of this study are available within the article.
